# Warmer and drier ecosystems select for smaller bacterial genomes in global soils

**DOI:** 10.1002/imt2.70

**Published:** 2023-01-03

**Authors:** Hongwei Liu, Haiyang Zhang, Jeff Powell, Manuel Delgado‐Baquerizo, Juntao Wang, Brajesh Singh

**Affiliations:** ^1^ Hawkesbury Institute for the Environment Western Sydney University Penrith New South Wales Australia; ^2^ College of Life Sciences Hebei University Baoding China; ^3^ Laboratorio de Biodiversidad y Funcionamiento Ecosistemico Instituto de Recursos Naturales y Agrobiología de Sevilla (IRNAS), CSIC Sevilla Spain; ^4^ Unidad Asociada CSIC‐UPO (BioFun) Universidad Pablo de Olavide Sevilla Spain; ^5^ Global Centre for Land‐Based Innovation Western Sydney University Penrith New South Wales Australia

**Keywords:** aridity, climate impacts, genome size, global soil, microbial trait, soil nutrients

## Abstract

Bacterial genome size reflects bacterial evolutionary processes and metabolic lifestyles, with implications for microbial community assembly and ecosystem functions. However, to understand the extent of genome‐mediated microbial responses to environmental selections, we require studies that observe genome size distributions along environmental gradients representing different conditions that soil bacteria normally encounter. In this study, we used surface soils collected from 237 sites across the globe and analyzed how environmental conditions (e.g., soil carbon and nutrients, aridity, pH, and temperature) affect soil bacterial occurrences and genome size at the community level using bacterial community profiling. We used a joint species distribution model to quantify the effects of environments on species occurrences and found that aridity was a major regulator of genome size with warmer and drier environments selecting bacteria with smaller genomes. Drought‐induced physiological constraints on bacterial growth (e.g., water scarcity for cell metabolisms) may have led to these correlations. This finding suggests that increasing cover by warmer and drier ecosystems may result in bacterial genome simplifications by a reduction of genome size.

## Highlights

- A joint species distribution model was used to quantify the environmental effects on occurrences of bacterial species.
- Warmer and drier environments favored bacteria with smaller genomes.
- Higher soil fertility (e.g., phosphorous) favored bacteria with smaller genomes.

## Graphical abstract

We used surface soils collected from 237 sites across the globe and analyzed how environmental conditions affect soil bacterial occurrences and genome size. We used a joint species distribution model to quantify the effects of environments on species occurrences and found that aridity was a major regulator of genome size with warmer and drier environments selecting bacteria with smaller genomes.

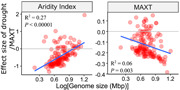

Trait‐based approaches have revolutionized ecology and provided demonstrable value to our ecological understanding (e.g., community assembly) of many taxa. However, knowledge of the importance of microbial traits lags behind other organisms (e.g., plants). In particular, we know little about the biogeographic pattern of microbial traits across the globe and the extent to which they are under environmental selection [1, 2]. Among microbial traits, genome size is fundamental for the prediction of microbial physiology, their ecological relationships with other microbes and the environment, and their evolutionary histories. This is because (i) genome size is linked to other bacterial traits related to life history strategies, for example, growth rate and tolerance to extreme conditions [3, 4], (ii) variation in genome size among microbial taxa can reflect evolutionary events such as genome expansion and reduction that may be associated with emerging of new species, functions or lifestyles [5, 6], and (iii) organisms with large genomes require more resources to grow and reproduce and can have a more restricted ecological distribution (large genome constraint hypothesis) [7]. In other words, smaller bacterial genomes require lower numbers of AGCT, and they can be more advantageous in nutrient‐limited (e.g., nitrogen, N and phosphorus, P) environments [7]. Therefore, the distribution of genomic traits in environments may exhibit unique patterns reflecting nutrient conditions.

Soil is a complex and heterogeneous ecosystem, and involves dynamic interactive bacterial communities that play key roles in regulating terrestrial nutrient cycling and plant productivity. Its function is strictly dependent on critical factors like water, light, and biodiversity, which provide the sustenance of humanity [8]. Recent studies have investigated microbial genome size in response to ecosystem type, trophic strategy, and oxygen use [9, 10], but investigations in soil microbial genomes and their responses to environmental factors along large spatial gradients are still limited. Current uncertainties in the biogeography of soil microbial genome size are due to two main reasons. First, investigations often focus on the relationship between a single environmental factor and genome size, yet to identify the most important environmental factors requires multifactorial investigation. For example, recent studies only investigated microbial genome size interactions with the environment in response to warming [11–13] and nutrient availability [14]. Second, these studies investigated only a narrow range of these environmental gradients, and research on ecosystems covering full ranges of climate, soil, and/or vegetation at a global scale is lacking. In theory, both a trade‐off (effects occur at both ends of the environmental gradients) and a unidirectional pattern (effects occur at one‐end of the gradients) can exist in scaled‐up/global scale environmental investigations [15]. Such environmental effects on microbial species occurrences can be either positive, negative, or neutral (Figure 1A), and the magnitude of the effect differs among bacterial species. To this end, four types of relationships between environmental effects and bacterial genome size are proposed: (i) trade‐offs (Figure 1B), (ii)–(iii) unidirectional effects (Figure 1C,D), and (iv) no correlations (Figure 1E). In this study, we aimed to investigate whether genome size could mediate the effects of environmental gradients on bacterial occurrences at the global scale. We hypothesized that environments with less favorable conditions such as drier soils (Figure 1F), warmer climates (Figure 1G), and reduced availability of soil nutrients (Figure 1H) favor occurrences of bacterial species with smaller genomes. These hypotheses are based on the large genome constraint hypothesis [6], and previous findings that nutrient limitation/drought‐induced physiological constraints on growth (e.g., water scarcity for cell metabolisms) may lead to genome streamlining (e.g., gene loss) [18].

**Figure 1:**
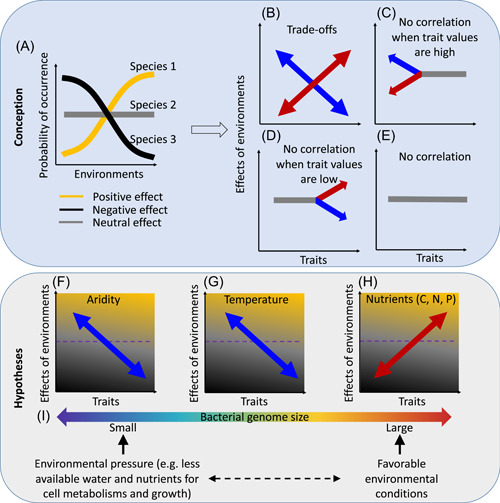
Conception and hypotheses. The ecological responses of the probabilities of bacterial occurrences to environmental gradients: positive (yellow), negative (black), and neutral (gray) (A). A positive (or negative) effect size indicates bacteria tend to occur with higher (or lower) probability when specific environmental conditions increase. An effect size is the slope of the relationship between bacterial occurrence probability and environmental conditions, calculated by beta parameters from a joint species distribution model [16, 17]. We provide four types of correlations between microbial traits and the effects size of environments. These are (i) trade‐offs where trait effects are bidirectional: the effect of the environmental gradients on the probability of species occurrence positively (red) or negatively (blue) shifts along the trait gradient (B), (ii) and (iii) the trait only exhibits an effect at one end of the environmental gradient, with only a unidirectional effect when trait values are low (C) or high (D); and (iv) no environmental effects across all trait levels (E). We hypothesized that shifting from favorable soil conditions to stressful environments (i.e., warmer and drier climates) favors bacteria with small GS in soil. In detail, we hypothesized that nutrients (carbon, nitrogen, and phosphorous) are positively associated with bacterial genome sizes (F), while maximum annual temperature (MAXT) (G) and aridity (H) are negatively associated with bacterial genome sizes. Overall, we hypothesized that stress conditions select bacteria with small genome sizes (I).

To test the above hypotheses, we analyzed bacterial communities of soil samples collected from 237 locations across the globe (Supporting Information: Figure S1), covering different ecosystem types (forests, grasslands, and shrublands) and diverse environmental gradients such as soil aridity, nutrients, and air temperature [19]. The soil properties showed a wide range of variations, with soil pH ranging from 4.04 to 9.21, soil C ranging from 0.15% to 34.77%, soil N ranging from 0.02% to 1.57%, soil P ranging from 75.10 to 4111.04 mg P kg^−1^ soil. These sites spanned a gradient of −11.4°C to 26.5°C mean annual temperature and 67–3085 mm mean annual precipitation. In total 135,175 soil bacterial features (2315 genera, sequence variants analyzed by QIIME2 software, http://qiime2.org/) were detected using 16S ribosomal RNA (rRNA) gene amplicon sequencing, and bacterial features annotated at the genus level were extracted (see Supporting Information methods, Figures S2, S3, and S4). Those unidentified and less identified bacterial genera, such as bacterium culture clones were removed from the genera feature table. To evaluate whether aggregation of these data at the genus level was appropriate, we surveyed bacterial genome size in the bacteria trait database [20], where data for both culturable and unculturable bacterial species are included for these genera. We found that across the species within most (95%) bacterial genera (*n* = 2238) had a low coefficient of variation (CV) (<20%) (Supporting Information: Figure S2, CV is defined as the ratio of the standard deviation to the mean for a certain genus). Further threshold sensitive analysis indicated that bacterial genome size estimation was robust for bacterial genera with CVs lower than 11% (this threshold was chosen based on sensitivity analysis, details in Supporting Information: Figure S3). Thus, we set a CV threshold of 11% across observations within a genus to select bacterial genera for inclusion in subsequent analyses, with 1237 bacterial genera being retained. We then matched our global soil microbiome data with these bacterial genera [20], resulting in 143 bacterial genera with genome size values, ranging from 1.58 Mbp (*Rickettsiella*) to 16.04 Mbp (*Minicystis*) (Supporting Information: Figure S4). Our selected genera included both abundant and rare genera (Supporting Information: Figure S5).

We then investigated relationships between the environmental gradients and bacterial genome size by fitting the data set with a joint species distribution model‐hierarchical modeling of species communities (HMSC) [16, 17] (Supporting Information: Figure S6). After model fitting and evaluation, we extracted the effect size, that is, the slope of the relationship between bacteria occurrence probability and environmental conditions, which was the beta‐parameters from HMSC with at least 95% posterior probability. For the correlation between genome size and the magnitude of environmental effects on bacterial occurrence probability, we performed phylogenetic least squares regression to account for phylogenetic autocorrelation among genera given that bacterial genome size is linked with their evolutionary history [21]. We found that the distance from the equator (absolute latitude) had a strong impact on genome size distribution in the soil (Figure 2A, *R* ^2^ = 0.22, *p* < 0.00001), in that bacteria with small genomes were more likely to have a positive association with increasing latitude while bacteria with large genomes exhibited the opposite. This result was in line with the geographical patterns previously reported [22], where the average‐genome size of bacterial communities decreased as distance from the equator increased.

**Figure 2:**
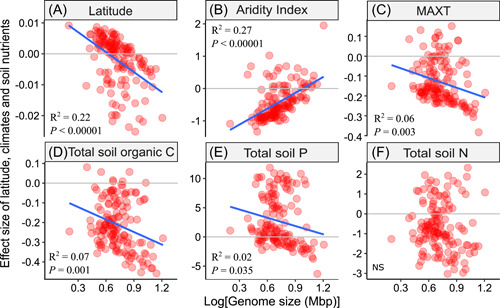
Correlations between bacterial genome size (log‐transformed; Mbp) and the effects of environmental conditions on bacterial occurrence. *Y* axis indicates effect sizes of an environmental condition, which are slopes (beta‐parameters) of the relationship between bacterial occurrence probability and environmental conditions, calculated from the Hierarchical Modeling of Species Communities (HMSC). A positive (or negative) effect size indicates bacterial genera tend to occur with higher (or lower) probability when specific environmental conditions increase. Solid blue lines indicate correlations being significant (*p* < 0.05) and were fitted considering phylogenetic correlations among bacteria genera. Aridity index reflects soil water availability (i.e., mean annual precipitation/evapotranspiration). MAXT, mean max annual temperature.

Importantly, we found that bacterial genome size significantly correlated with aridity (Figure 2B, *R* ^2^ = 0.27, *p* < 0.00001), in that bacteria with small genomes exhibited positive responses to increasing aridity (negative response to an increasing aridity index) while bacterial genera with large genomes tended to not exhibit any response to aridity (the effect sizes related these bacteria were close to zero) (Figure 2B). This trend of bacterial selection with small genomes was consistent across different taxonomic levels such as the order and phylum level (Supporting Information: Figure S7). Our further analyses demonstrated that large genome‐sized bacterial species contain a larger number of genes (Supporting Information: Figure S6A, *R* ^2^ = 0.97, *p* < 0.00001) (Supporting Information: Figure S8A), which suggests that a genome size reduction in arid ecosystem may result in bacterial genome simplification. Consistently, a recent study also reported a positive correlation between bacterial community weight mean genome size with mean annual precipitation [23]. However, it is unclear in their study whether the positive correlation was caused by the bacteria with large genome sizes being positively selected by increasing water availability or bacteria with small genome sizes being excluded by increasing water availability, or both. Our results support the second scenario, where bacterial species with small genome sizes had more negative responses to increased water availability than those with large genome size species. Similarly, warmer climates, as indicated by higher mean maximum temperature (MAXT), favored the occurrence of bacteria with small genome size by excluding large genome‐sized bacteria (Figure 2C, *R* ^2^ = 0.06, *p* = 0.003). This result is consistent with findings in previous reports [12, 24], and supported by our further analyses, which showed large genome‐sized bacterial species had a lower optimum temperature (Supporting Information: Figure S8B, *R* ^2^ = 0.07, *p* = 0.001). This means that an increased temperature can induce higher selective pressures on bacteria with large genome sizes than on bacteria with small genome sizes.

We also found that bacterial genome sizes weakly but significantly correlated with soil carbon (C, Figure 2D, *R* ^2^ = 0.07, *p* = 0.001) and P (Figure 2E, *R* ^2^ = 0.02, *p* = 0.035) concentrations, in that bacteria with small genomes exhibited less negative responses to increasing soil C or more positive responses to increasing soil P, suggesting that higher soil fertility favors bacteria with smaller genomes. Soil nitrogen did not exhibit a significant correlation (Figure 2F). While contradicting our hypothesis (based on the large genome constraint theory), our results are supported by previous studies where enrichments of soil nutrients (e.g., P) decreased the mean genome sizes of bacteria at the community level in grassland soil [14]. These findings suggest that larger genome‐sized bacterial species can dominate in environments where resources are scarce but diverse, and where there is little penalty for slow‐growth soil bacteria, in terms of their colonization and competition in soils [22, 25]. It would be also important to investigate how genome size responses to soil nutrient availability are linked with their r/K strategy in future studies. For example, previous studies showed that soil bacteria from copiotrophic (r strategy) Firmicutes increased in relative abundance by nutrient addition while oligotrophic (K strategy) bacteria such as Actinobacteria decreased in abundance [26]. Consistently, we found that Firmicutes had relatively small and Actinobacteria had large genomes (Supporting Information: Figure S5), and, therefore, our results (higher soil fertility favors bacteria with small genomes) can be explained by the r/K selection theory [27]. We also found that genome size did not affect the sensitivity of bacterial occurrence probability to changes in soil pH and mean minimum annual temperature (Supporting Information: Figure S9). By contrast, bacterial genome size significantly correlated with net primary production (NPP, the carbon amount retained by plants or other primary producers) and ultraviolet (UV) light, in that bacteria with large genomes exhibited positive correlations to increasing NPP and UV light while bacterial genera with small genomes tended to not exhibit any correlations with NPP and UV light (Supporting Information: Figure S9). Increasing NPP favored bacteria with large genomes was in line with our results that warming and drier ecosystems selected for smaller bacterial genomes, because NPP is generally lower under stressed environments (e.g., drought). A positive response to UV light intensity also indicates that large genome‐sized bacteria are more tolerant to strong UV light. Mechanisms for these findings are still unclear and require future studies to investigate in‐depth.

We acknowledge that the community‐weighted trait means (CWM), which is the average of trait values for species at each site weighted by species abundance, is also commonly used to study trait–environment relationships. However, we did not observe significant patterns between CWM genome size and environmental gradients (Supporting Information: Figure S10), which may highlight the strength of the joint species distribution approach in detecting soil bacterial responses to environmental changes. Recent studies indicated that the model‐based approach outperforms the CWM‐based approaches in terms of results accuracy and statistical power [28, 29].

Overall, our study provides novel evidence that environmental stress associated with increases in aridity and warmer climates favors bacteria with smaller genomes in soils. Drought‐induced physiological constraints on growth (e.g., water scarcity for cell metabolisms) may have led to these correlations. Bacterial community change can lead to changes in soil ecological processes such as carbon sequestration. For example, previous studies showed that bacteria with small genomes are associated with high carbon use efficiency (CUE, i.e., the ratio of net carbon gain to gross carbon assimilation, a key factor that controls carbon sequestration) [30]. Drought stress reduces plant carbon assimilation and availability to soil microbes. Shifting to a small genome‐size‐dominated community is important to increase CUE at the community level and help the ecosystem to maintain functions under stressful conditions. Favoring bacterial species with small genome sizes may also have consequences on the redundancy of microbial functions and resilience. Soil microbes make up a complex gene pool; reducing or discarding genes for redundant metabolic burdens may be a strategy for microbes to survive stresses [13]. Now we are facing climate change that is more rapid than ever before. Despite the unknown consequences of such microbial changes to ecosystem functions, future climate change research and policy‐making should consider soil bacteria and their functional traits as they play important roles in soil carbon sequestration and maintaining ecosystem functions. Our study also suggests that lower soil fertility (soil C and P) and higher UV light levels favor bacteria with larger genomes, which can dominate in soils where resources are scarce but without major restrictions on slow‐growing bacteria. These findings point toward fundamental mechanisms underpinning nucleotide selection in bacterial communities in global soils. The knowledge is also critical for efforts to predict biogeochemical feedback in the future climate system, where drought events are expected to occur more frequently and to last longer at a global scale. Our study employed bacterial analyses at the genus level by obtaining genome size data from a publicly available database. This method is reliable in testing our hypothesis, and it provides a useful tool to investigate soil genomes based on amplicon sequencing that is still vastly used for microbiome research. It has shortfalls in that only a part of the soil bacterial community can be included in analyses due to the inherent limitations of 16S rRNA gene amplicon sequencing in profiling bacterial communities (e.g., 16S rRNA gene is conserved and it may mask certain trait variations within bacterial communities). Future research is warranted to validify our findings by investigating bacterial community assembly in soil and other ecosystems (e.g., marine ecosystems) by determining genome size from metagenomes across environmental gradients.

## AUTHOR CONTRIBUTIONS

Hongwei Liu, Haiyang Zhang, Brajesh Singh, and Manuel Delgado‐Baquerizo conceived the idea, and Haiyang Zhang, Hongwei Liu, Jeff Powell, and Juntao Wang analyzed the data. Hongwei Liu and Haiyang Zhang wrote the paper and all authors have significantly contributed to the manuscript revision.

## CONFLICT OF INTEREST

The authors declare no conflict of interest.

## Supporting information

Supporting information.

## Data Availability

The data that supports the findings of this study are available in the supplementary material of this article. 16S rDNA data and metadata used in this study are available in Figshare (DOI: 10.6084/m9. figshare.5611321). Supporting Information materials (figures, tables, scripts, graphical abstract, slides, videos, Chinese translated version, and update materials) may be found in the online DOI or iMeta Science http://www.imeta.science/.
