# Supplementary material for: Warmer and drier ecosystems select for smaller bacterial genomes in global soils
Source: Imeta. 2023 Jan 3;2(1):e70. doi: 10.1002/imt2.70 (PMC10989973; doi:10.1002/imt2.70)
Supplement: Supplementary file 1 — Supporting information. [file IMT2-2-e70-s001.docx]

**Supplementary Materials**

*Soil samples, collection, traits, and global distribution*

We analysed soil bacterial communities collected from 237 locations across six continents (Figure S1), which have been previously used for bacterial analyses at a global scale [1]. All samples collected were shipped to the Universidad Rey Juan Carlos (Spain) for laboratory analyses. At each site, the coordinates for soil collection were recorded *in situ* with a portable GPS, and a soil sample was collected from the top ~7.5 cm under the most common vegetation. Two sub-samples were then obtained, with one being frozen at -20ºC for molecular analyses and the other for physio-chemical analyses. For all samples, we measured soil pH (using a pH meter, in a 1: 2.5 mass: volume soil and water suspension), soil texture (% of fine fractions: clay + silt), total soil organic carbon, soil nitrogen and phosphorus concentrations using standard laboratory methods [1]. The soil properties showed a wide range of variations, with soil pH ranging from 4.04 to 9.21, soil C ranging from 0.15% to 34.77%, soil N ranging from 0.02% to 1.57%, soil P ranging from 75.10 to 4111.04 mg P kg^-1^ soil, and fine texture fraction (% clay + silt) ranging from 1.40% to 92.00%. These sites where samples were collected contained ecosystem types of forests, grasslands and shrublands, and across climatic regions of arid, temperate, tropical, continental and polar ecosystems. The plots spanned a gradient of -11.4 to 26.5ºC mean annual temperature and 67 to 3,085 mm mean annual precipitation.

The maximum and minimum temperature, seasonal precipitation for all sampling locations were obtained from the WorldClim database (1 km resolution, [www.worldclim.org](http://www.worldclim.org)). The Aridity Index, calculated by precipitation/evapotranspiration was obtained from the Global Potential Evapotranspiration database (https://cgiarcsi.community/data/global-aridity-and-pet-database/). Aridity index is a better measure of the long-term water availability than precipitation at each sampling location because it considers both mean annual precipitation and potential evapotranspiration. Ultraviolet index (UV index, 0-16) that measures UV radiation intensity was retrieved from the NASA's Aura satellite (50 km resolution, https://neo.sci.gsfc.nasa.gov). The Normalised Difference Vegetation Index (NDVI) data were obtained from the Moderate Resolution Imaging Spectroradiometer (MODIS) aboard NASA's Terra satellites (<http://neo.sci.gsfc.nasa.gov/>), which was used as a global measure of vegetation greenness and distribution across the global landscapes and a proxy for net plant primary productivity (NPP). The monthly average value for this variable was calculated between the 2003-2015 periods, which has a resolution of about 10 Km.

*Bioinformatics for analysing soil bacterial community and genus screening*

Bacterial community profiling was performed by amplicon sequencing using 16S rRNA gene primers 341F (CCTACGGGNGGCWGCAG) and 805R (GACTACHVGGGTATCTAATCC). The sequencing was conducted at the Western Sydney University Next Generation Sequencing Facility as per the standard MiSeq sequencing procedures. All raw sequencing files were imported to QIIME2 software (version 2020.2; <http://qiime2.org/>) using a python code. DADA2 v2019.7.0 [2] was used to remove primer sequences, correct errors, remove chimera and merge the forward and reverse sequences using as per default parameters. The amplicon sequence variants (ASVs) obtained were summarised and assigned with taxonomic information using an RDP classifier pre-trained on Silva 138, 99% OTUs full-length sequences. Sequences including chloroplast, mitochondria, eukaryota, archaea and unassigned were removed from feature table using the feature-table filter-features command. Hereafter, to accommodate matching to the bacterial trait database, we collapsed features in the feature table at the genus level. Those unidentified and less identified bacterial genera, such as bacterium culture clone and metagenome-like genera were removed from the genera feature table, prior to estimation of bacterial genome sizes.

*Estimation of genome size, coding gene number and other traits.*

Bacterial features annotated at the genus level were extracted because there was limited coverage for features annotated at the species level or below. To evaluate whether aggregation of these data at the genus level was appropriate, we surveyed bacterial genome size in the bacteria trait database, where data for both culturable and unculturable bacterial species are included [3] for these genera and found that across the species within most (95%) bacterial genera (n = 2,238) had a low coefficient of variation (CV) (<20%) (Figure S2). Further threshold sensitive analysis indicated that bacterial genome size estimation was robust for bacterial genera with CVs lower than 11% (details in Figure S3). Thus, we set a CV threshold of 11% across observations within a genus to select bacterial genera for inclusion in subsequent analyses, with 1,237 bacterial genera being retained. We then matched our global soil microbiome data with these bacterial genera [3], resulting in 143 bacterial genera with genome size values, ranging from 1.58 Mbp (*Rickettsiella*) to 16.04 Mbp (*Minicystis*) (Figure S4).

*Species occurrence along environmental gradients tested by the joint species distribution model*

To test how environmental conditions affect the probability of bacteria occurrence and whether genome size explains species occurrences by their interactions with environmental gradients, we fitted our dataset with a joint species distribution model - Hierarchical Modelling of Species Communities (HMSC). HMSC is a multivariate generalized linear mixed modelling framework [4, 5]. It is primarily developed for community ecology and modelled the response matrix (Y, species occurrences) on a set of sampling units (e.g. spatial locations). The environmental predictors’ matrix (X) contains, for instance, climate gradients in the sampling units, and the species trait matrix (T) includes traits such as genome size. We fitted the HMSC model with the R-package Hmsc [5]. The ten environmental predictors included in our HMSC model are: latitude, NPP averaged from 2003-2015, aridity index, MAXT, MINT, soil C, N, P concentration, pH, and UV light. These factors were included as fixed effects (i.e. predictors), and the sampling unit (soil samples) as the random effect. We generated species occurrence data from DNA sequencing data at the genus level. After matching the sequencing data with our genome size database, we filtered the data by two additional steps. First, samples were excluded if they contained less than 20 bacterial genera in one community (i.e. soil sample). Second, species were excluded if they occurred in less than 5 soil samples. These steps were implemented to avoid biased influences from rare bacterial genera on model fitting.

In brief, the occurrence probability of each bacterial genus for each soil sample was fitted with probit regression (presence-absence) against the ten standardized environmental predictors. Then the model-derived beta-parameter for each environmental predictor X reveals how certain bacterial genera responded to predictor X. We fitted the models with four Markov Chain Monte Carlo (MCMC) chains, each of which consisted of 39,000 iterations, out of which we discarded the first 9,000 as the burn-in and thinned the remaining by a factor of 150, thus yielding 200 samples per chain and 800 samples in total. We assessed the convergence of the MCMC chains by examining the effective sample sizes and the distribution of the potential scale reduction factor over the parameters related to the fixed effects (beta–parameters), equivalent to the Gelman-Rubin statistic. Our results showed that the MCMC convergence of the HMSC models was satisfactory (Figure S6). After model fitting and evaluation, we extracted the effect size, i.e. the slope of the relationship between bacteria occurrence probability and environmental conditions, which was the beta-parameters from HMSC with at least 95% posterior probability. Then the correlations between effect size and bacteria GS at the genus level were fitted with *phylolm* from R package phylolm to account for shared evolutionary histories among genera [6]. We reported the trait-environments correlations for latitude, climate and soil nutrients in the main text while supplied results for MINT, pH, NPP and UV light in the supplementary material for simplicity (Figure S9). Note that estimation of beta parameter from HMSC represented the sensitivity of species occurrence to the changes in environmental conditions. HMSC uses the model-based approach to estimate the trait-environmental correlations. By contrast, the community-weighted trait means (CWM), which is the average of trait values for species at each site weighted by species abundance, is also commonly used to study trait-environment relationships. Recent studies indicated that the model-based approach outperforms the CWM-based approaches in terms of statistics’ sampling accuracy and statistical power [7, 8]. Here we reported results from CWM in the supplementary material for comparison (Figure S10). All above analyses were conducted in R software 4.0 [9].


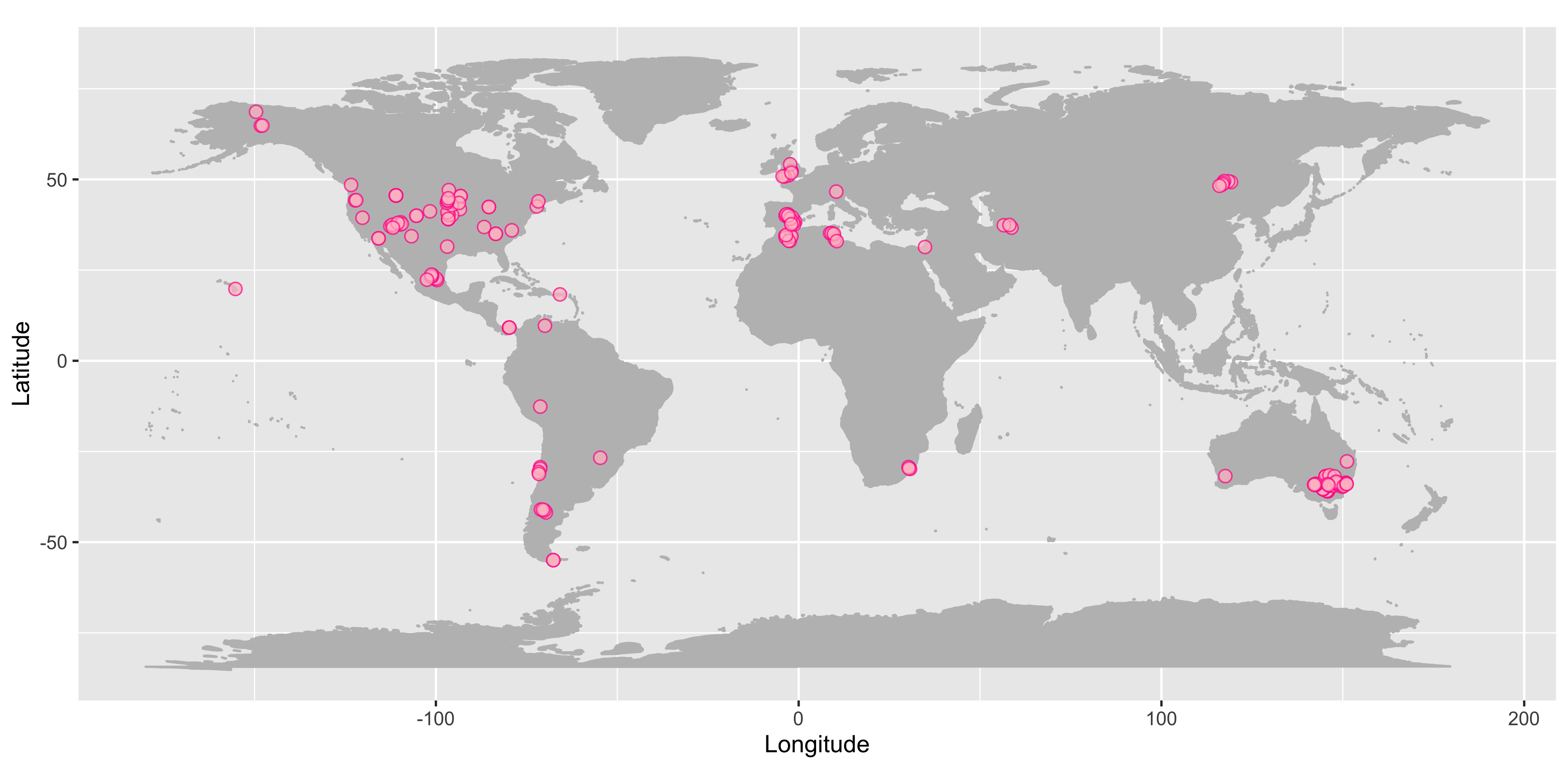


**Figure S1** A map summarising sampling locations of the 237 samples across the globe. Each dot represents a sampling location.

**
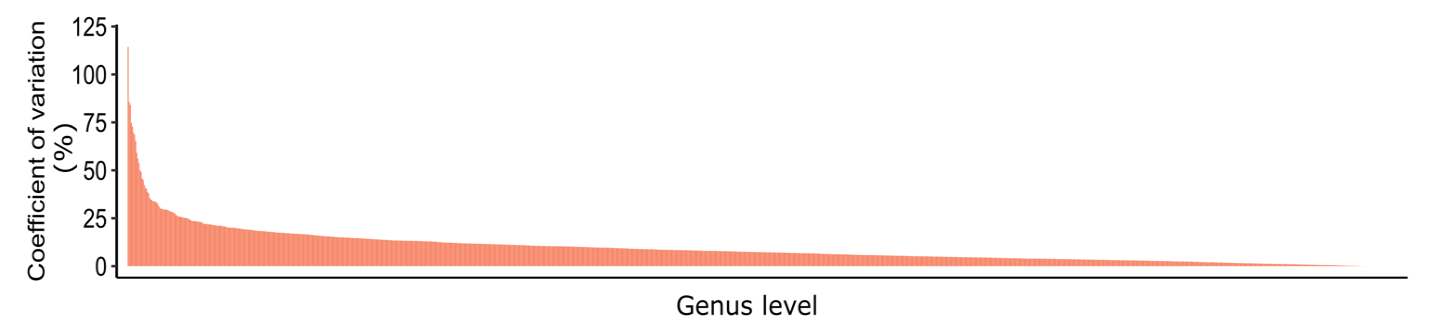
Figure S2** Coefficient of variation (CV, %) of genome size from the bacterial trait database at the genus level (n = 2,238), with these bacterial genera generally having relatively low CV. Each red bar represents a genus, ordered by their CV values along the x-axis.

**
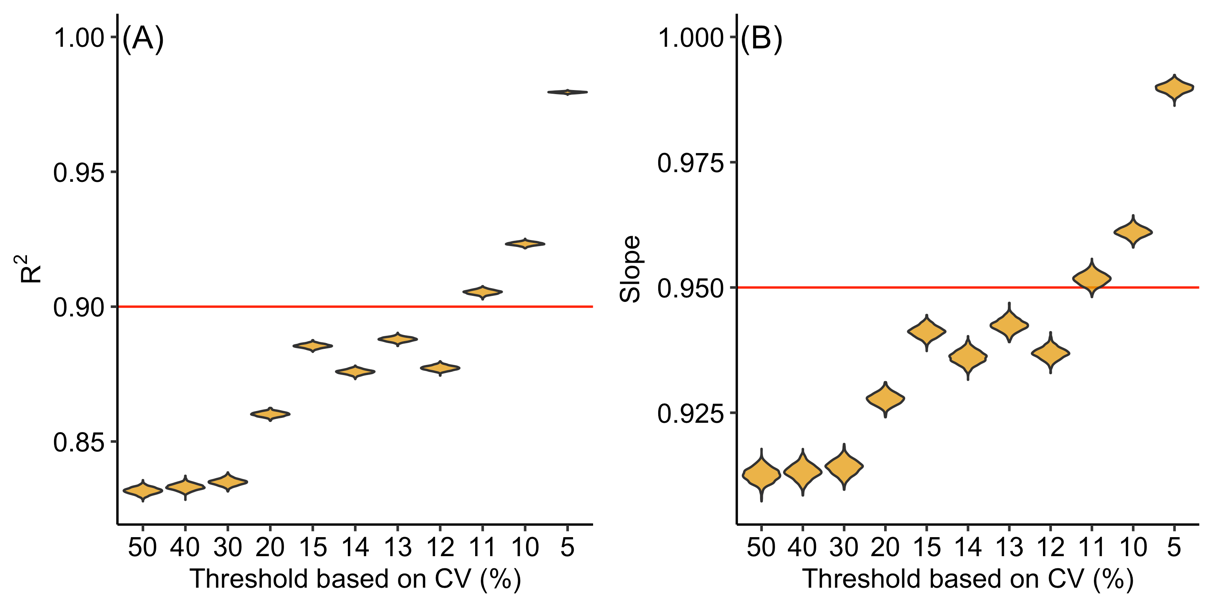
**

**Figure S3**. Violin plots demonstrate R^2^ extracted from the correlation between predicted genome size and true genome size value at the species level for each threshold that was used to filter the bacterial genera. Since we used the mean genome size value at the genus level for our model analysis, we only included the genera that were conservative in genome size values within each genus. To do so, we first filtered the genera by keeping genera if their CV was lower than a certain threshold. We repeated this filtering process with a series of thresholds: 5%, 10%, 11%, 12%, 13%, 14%, 15%, 20%, 30%, 40% and 50% (A). After each filtering, we then randomly generated the predicted genome size value for each species from a normal distribution, with the mean and standard deviation (SD) of the normal distribution being derived from the GS database for each genus. Then the predicted genome size vs. true genome size was compared using a linear model (lm) and extracted the R^2^ (A) and slope (B). We repeated this prediction process 999 times for getting the R^2^ values for each threshold. The outcome results suggested that the lower the CV threshold we chose, the higher predictor power we gain (i.e., R^2^ and slope close to 1). The Figures (A and B) indicated that when we chose CV < 11%, we could manage the R^2^ larger than 0.90 while slope was close to 1.0 (> 0.95), which means the predicted value based on genome size mean and SD can be quite close to true values.

**
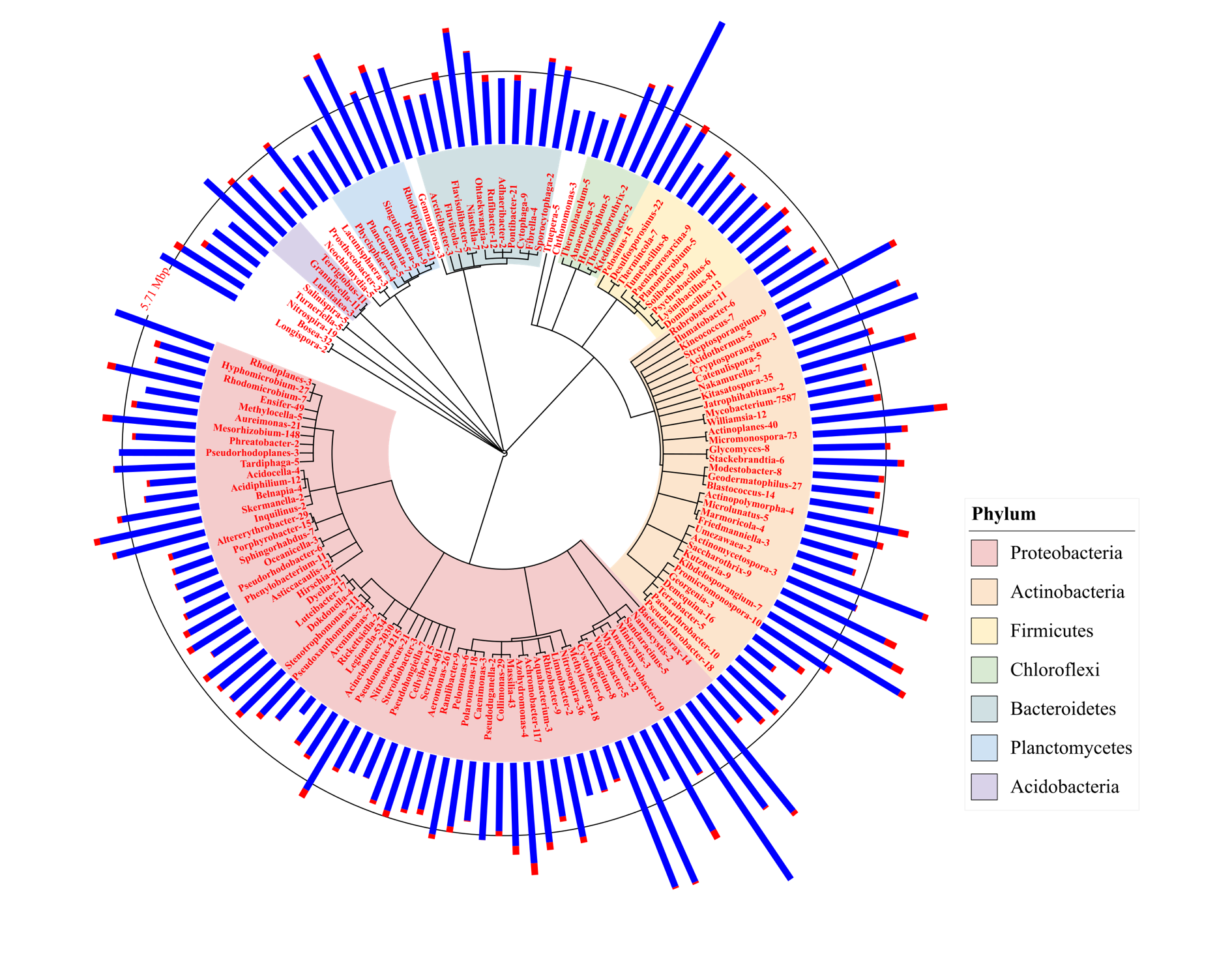
**

**Figure S4** Phylogenetic tree with terminal branches for the studied bacteria genus. Blue bars show the genome size for each genus. Red bars represent standard deviation (SD) of genome size of that genus. The number of available genomes of a specific genus is labelled beside its name. Black circle line indicates the mean genome size averaged for all genera, which is 5.71 Mbp.


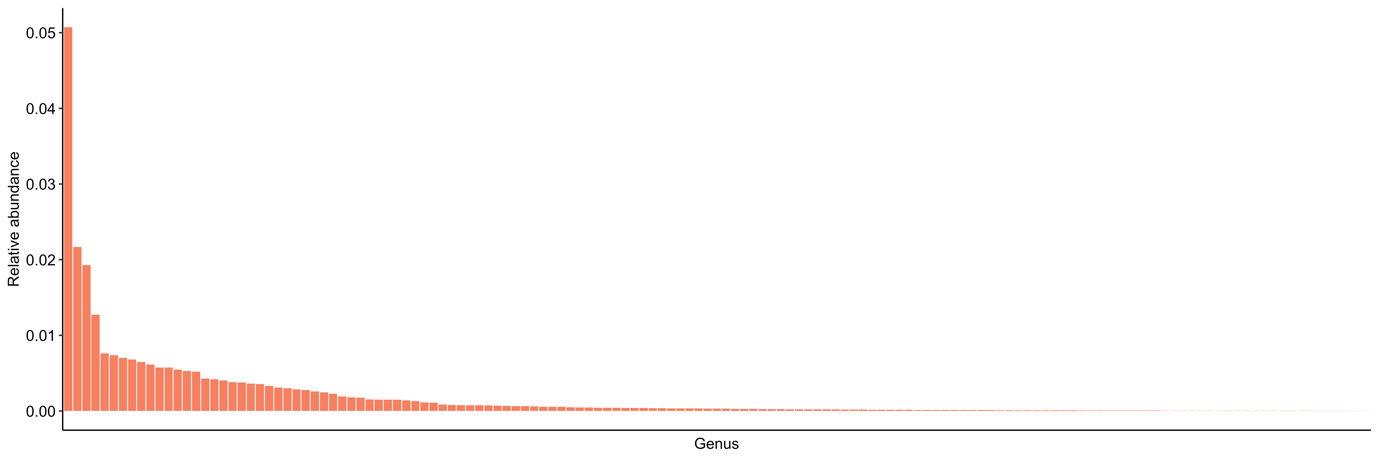


**Figure S5** The relative abundance of each of the bacterial genera included in the joint species distribution model.

**
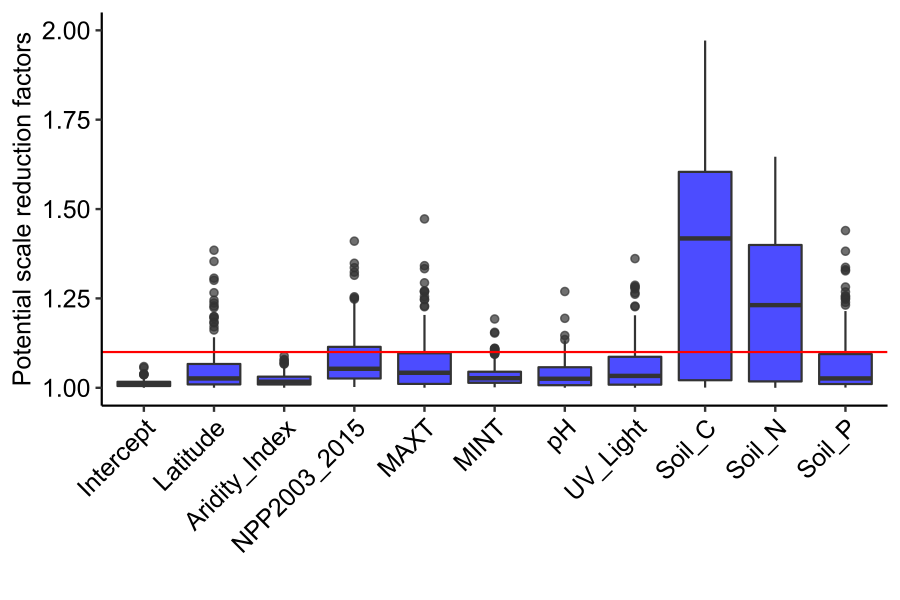
**

**Figure S6** Markov Chain Monte Carlo (MCMC) convergence statistics after the Hierarchical Modelling of Species Communities (HMSC) fitting, measured as potential scale reduction factors. Each boxplot shows the distribution of values over the species-specific beta-parameters for models that include the intercept, latitude, net primary production (NPP, averaged for each site from year 2003 to 2015), mean max annual temperature (MAXT), aridity, mean min annual temperature (MINT), soil pH, ultraviolet (UV) light, soil carbon (C), nitrogen (N), and phosphorus (P) as environmental predictors. The beta-parameters represent the response of the species to the included covariates. As the majority of the potential scale reduction factors are close to 1.00 (lower than 1.1; red line), we concluded that the MCMC convergence was good.


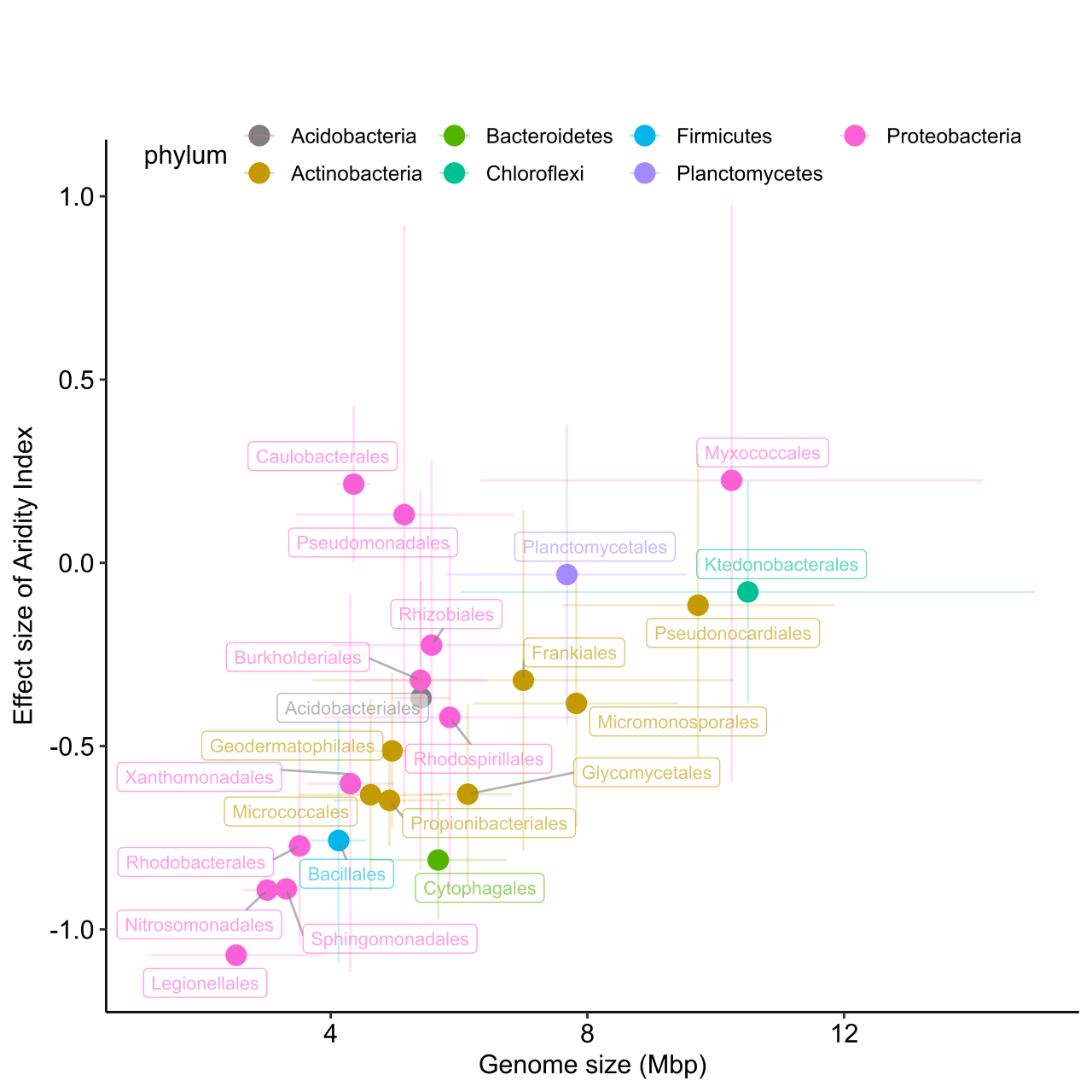


**Figure S7** Distribution of effect size of aridity index against bacterial genome size at the bacterial order level. Effect sizes represent the magnitude of species occurrence in response to the increasing aridity index. Each dot represents a bacterial order observed in this study, and their taxonomy at the phylum level was labelled on top of the graph. Cross lines represent the standard deviation when averaging values for all genera for each order.

**
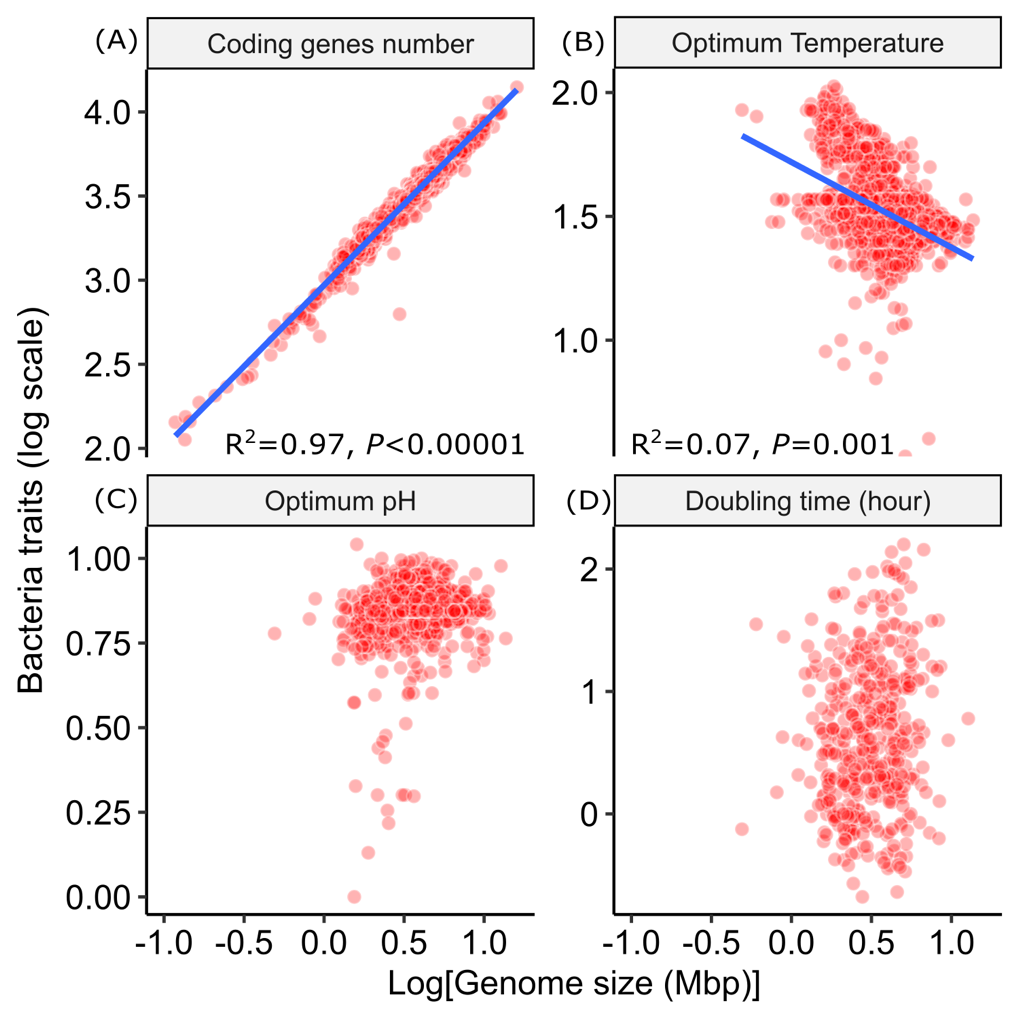
**

**Figure S8** Correlations between genome size (log-transformed; Mbp) and bacteria traits at the genus level. The traits included coding gene number (A), optimum temperature (B), optimum pH (C) and bacteria doubling time that indicates bacterial growth rate (D). Blue lines indicate significant correlations (*P* < 0.05) that were fitted considering phylogenetic correlations among bacterial genera. Data for plotting the correlations were from the bacterial and archaeal phenotypic trait database [3].

**
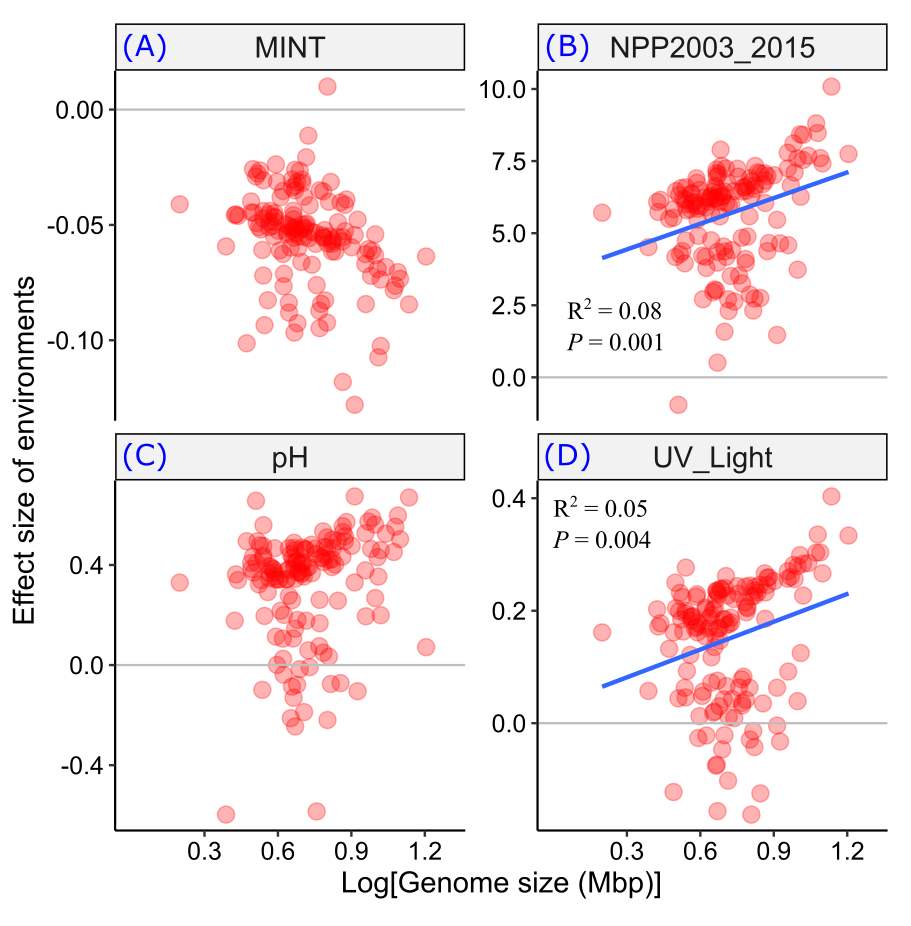
**

**Figure S9** Correlations between genome size (log-transformed; Mbp) and the effect size of environmental conditions on the probability of bacterial occurrence. The tested environmental factors were (A) MINT, (B) NPP2004_2015, (C) pH, and (D) UV_Light. Effect size is the slope of the relationship between bacterial occurrence probability and environmental conditions, which was the beta-parameters (i.e. slopes) from the Hierarchical Modelling of Species Communities (HMSC). A positive (or negative) effect size indicates bacterial genera tend to occur with higher (or lower) probability when specific environmental conditions increase. MINT: mean minimum annual temperature. NPP2003_2015: net primary production averaged for each site from year 2003 to 2015.


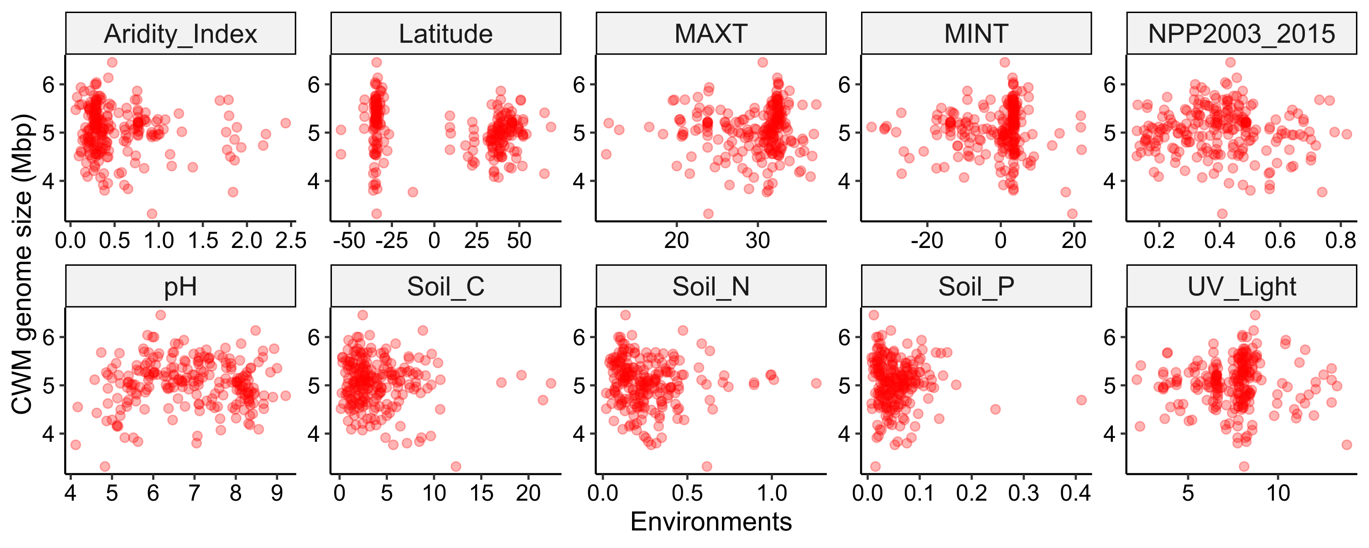


**Figure S10** Community weighted mean (CWM) for bacterial genome size in soil relative to environmental variables. MAXT/MINT: mean maximum/minimum annual temperature. NPP2003_2015: net primary production averaged for each site from year 2003 to 2015. These results did not reveal significant patterns between CWM genome size and environmental gradients, which may highlight the strength of the joint species distribution approach in detecting soil bacterial responses to environmental changes.

**References**

1. Delgado-Baquerizo, Manuel, Angela M. Oliverio, Tess E. Brewer, Alberto Benavent-González, David J. Eldridge, Richard D. Bardgett, Fernando T. Maestre, Brajesh K. Singh, Noah Fierer. 2018. “A global atlas of the dominant bacteria found in soil.” *Science* 359: 320-325. <https://doi.org/10.1126/science.aap9516>

2. Callahan, Benjamin J., Paul J. McMurdie, Michael J. Rosen, Andrew W. Han, Amy Jo A. Johnson, Susan P. Holmes. 2016. “DADA2: High-resolution sample inference from Illumina amplicon data.” *Nat Methods* 13: 581-583. <https://doi.org/10.1038/nmeth.3869>

3. Madin, Joshua S., Daniel A. Nielsen, Maria Brbic, Ross Corkrey, David Danko, Kyle Edwards, Martin K. M. Engqvist, et al. 2020. “A synthesis of bacterial and archaeal phenotypic trait data.” *Sci Data* 7: 170. <https://doi.org/10.1038/s41597-020-0497-4>

4. Ovaskainen, Otso, Gleb Tikhonov, Anna Norberg, F. Guillaume Blanchet, Leo Duan, David Dunson, Tomas Roslin, Nerea Abrego. 2017. “How to make more out of community data? A conceptual framework and its implementation as models and software.” 20: 561-576. <https://doi.org/10.1111/ele.12757>

5. Tikhonov, Gleb, Øystein H Opedal, Nerea Abrego, Aleksi Lehikoinen, Melinda MJ de Jonge, Jari Oksanen, Otso Ovaskainen. 2020. “Joint species distribution modelling with the r‐package Hmsc.” *Methods Ecol Evol* 11: 442-447. <https://doi.org/10.1111/2041-210X.13345>

6. Tung Ho, Lam si, Cécile Ané. 2014. “A linear-time algorithm for Gaussian and non-Gaussian trait evolution models.” *Syst Biol* 63: 397-408. <https://doi.org/10.1093/sysbio/syu005>

7. Peres-Neto, Pedro R., Stéphane Dray, Cajo J. F. ter Braak. 2017. “Linking trait variation to the environment: critical issues with community-weighted mean correlation resolved by the fourth-corner approach.” *Ecography* 40: 806-816. https://doi.org/10.1111/ecog.02302

8. Miller, Jesse E. D., Ellen I. Damschen, Anthony R. Ives. 2019. “Functional traits and community composition: A comparison among community-weighted means, weighted correlations, and multilevel models.” *Methods in Ecology and Evolution* 10: 415-425. https://doi.org/10.1111/2041-210X.13119

9. Team, R Core. 2013. “R: A language and environment for statistical computing.” R Foundation for Statistical Computing, Vienna, Austria. Available online at <https://www.R-project.org/>.
